# Glucose-6-Phosphate Dehydrogenase, ZwfA, a Dual Cofactor-Specific Isozyme Is Predominantly Involved in the Glucose Metabolism of Pseudomonas bharatica CSV86^T^

**DOI:** 10.1128/spectrum.03818-22

**Published:** 2022-11-10

**Authors:** Bhavik A. Shah, Sravanti T. Kasarlawar, Prashant S. Phale

**Affiliations:** a Department of Biosciences and Bioengineering, Indian Institute of Technology-Mumbai, Mumbai, India; Swansea University

**Keywords:** glucose-6-phosphate dehydrogenase, Zwf, isozymes, transcription analysis, metabolic flexibility, redox cofactors, dual cofactor specificity, kinetic characterization, cooperativity

## Abstract

Glucose-6-phosphate dehydrogenase (Zwf) is an important enzyme in glucose metabolism via the Entner-Doudoroff pathway and the first enzyme in the oxidative pentose-phosphate pathway. It generates NAD(P)H during the conversion of glucose-6-phosphate (G6P) to 6-phosphogluconolactone, thus aiding in anabolic processes, energy yield, and oxidative stress responses. Pseudomonas bharatica CSV86^T^ preferentially utilized aromatic compounds over glucose and exhibited a significantly lower growth rate on glucose (0.24 h^−1^) with a prolonged lag phase (~10 h). In strain CSV86^T^, glucose was metabolized via the intracellular phosphorylative route only because it lacked an oxidative (gluconate and 2-ketogluconate) route. The genome harbored three genes *zwf*A, *zwf*B, and *zwf*C encoding three Zwf isozymes. The present study aimed to understand gene arrangement, gene expression profiling, and molecular and kinetic properties of the purified enzymes to unveil their physiological significance in the strain CSV86^T^. The *zwf*A was found to be a part of the *zwf*A-*pgl*-*eda* operon, which was proximal to other glucose transport and metabolic clusters. The *zwf*B was found to be arranged as a *gnd*-*zwf*B operon, while *zwf*C was present independently. Among the three, *zwf*A was transcribed maximally, and the purified ZwfA displayed the highest catalytic efficiency, cooperativity with respect to G6P, and dual cofactor specificity. Isozymes ZwfB and ZwfC were NADP^+^-preferring and NADP^+^-specific, respectively. Among other functionally characterized Zwfs, ZwfA from strain CSV86^T^ displayed poor catalytic efficiency and the further absence of oxidative routes of glucose metabolism reflected its lower growth rate on glucose compared to P. putida KT2440 and could be probable reasons for the unique carbon source utilization hierarchy.

**IMPORTANCE** Pseudomonas bharatica CSV86^T^ metabolizes glucose exclusively via the intracellular phosphorylative Entner-Doudoroff pathway leading the entire glucose flux through Zwf as the strain lacks oxidative routes. This may lead to limiting the concentration of downstream metabolic intermediates. The strain CSV86^T^ possesses three isoforms of glucose-6-phosphate dehydrogenase, ZwfA, ZwfB, and ZwfC. The expression profile and kinetic properties of purified enzymes will help to understand glucose metabolism. Isozyme ZwfA dominated in terms of expression and displayed cooperativity with dual cofactor specificity. ZwfB preferred NADP^+^, and ZwfC was NADP^+^ specific, which may aid in redox cofactor balance. Such beneficial metabolic flexibility facilitated the regulation of metabolic pathways giving survival/fitness advantages in dynamic environments. Additionally, multiple genes allowed the distribution of function among these isoforms where the primary function was allocated to one of the isoforms.

## INTRODUCTION

**FIG 1:**
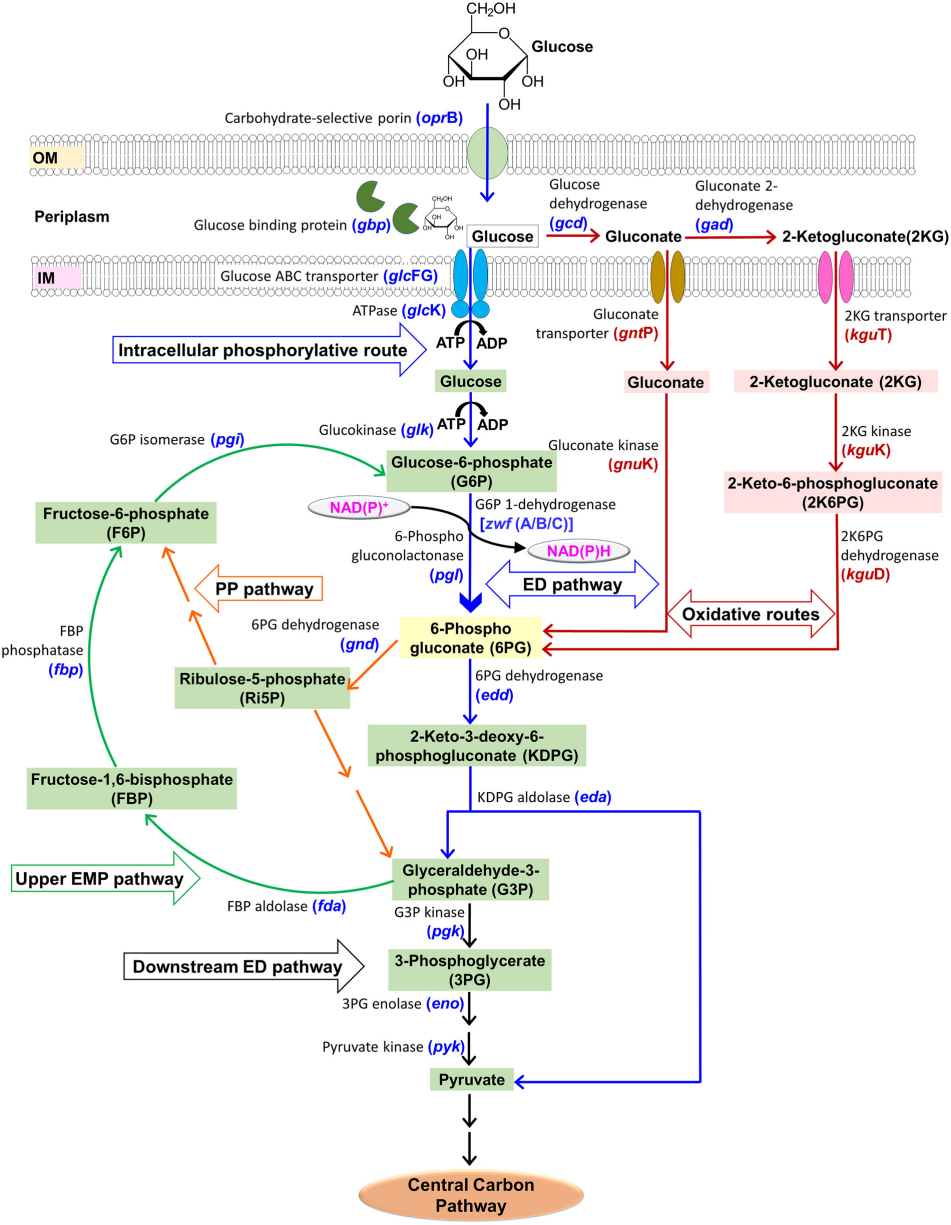
Glucose metabolic pathway in Pseudomonas spp. ATP-dependent glucose transport and intracellular phosphorylation route are depicted by blue color arrows, oxidative routes (gluconate and 2-ketogluconate routes, which were absent in strain CSV86^T^) are depicted by red color arrows, the upper EMP pathway by green color arrows, the downstream ED pathway in black color arrows while PP pathway is depicted by orange color arrows.

Glucose metabolism is one of the key metabolic pathways evolved in bacteria to yield energy and biomass. There are two major routes for glucose metabolism, the Embden-Meyerhof-Parnas (EMP) and the Entner-Doudoroff (ED) pathway (1). In Escherichia coli and Bacillus subtilis, glucose is channelized mainly via the EMP pathway. However, in Pseudomonas spp., glucose is metabolized through the ED pathway because they lack the key glycolytic enzyme, 6-phosphofructokinase (6pfk) required for the functional EMP pathway (2). Compared to the EMP pathway, glucose metabolism via the ED pathway yields a half amount of ATP. However, it is economically balanced by fewer metabolic steps, thus offering a beneficial trade-off for enzyme synthesis versus energy yield (3, 4). In Pseudomonas spp. glucose is transported and metabolized majorly (80% to 90%) through the oxidative (gluconate and 2-ketogluconate) route while the remaining is metabolized through ATP-dependent glucose transport followed by intracellular phosphorylation pathway. Metabolic steps further involved NAD(P)H production by the action of glucose-6-phosphate dehydrogenase (Zwf or G6PDH) (Fig. 1) (5–8). All metabolic routes converge at 6-phosphogluconate (6PG). Organisms also followed the cyclical ED-EMP pathway where glucose-6-phosphate (G6P) generated via the gluconeogenic EMP route (upper EMP pathway) was funneled into the ED pathway to increase NAD(P)H production (Fig. 1) (9, 10). Zwf is an important enzyme in ED as well as the pentose phosphate (PP) pathway. The conversion of G6P to 6PG in the PP and ED pathways share common metabolic steps. In the PP pathway, 6PG was metabolized to ribulose-5-phosphate by decarboxylating 6-phosphogluconate dehydrogenase (encoded by *gnd*) along with the generation of NADPH (Fig. 1). The PP pathway is majorly responsible to produce essential metabolites/anabolic precursors, including C4, C5, and C7 sugars and reducing equivalent NADPH. Thus, during glucose metabolism, the redox-cofactor balance is maintained by flux distribution between Zwf (phosphorylative ED, PP) and non-Zwf (EMP, oxidative ED) routes (Fig. 1).

In Escherichia coli, the Zwf is majorly involved in the PP pathway while the ED pathway remains inactive (11). The deletion of glucose-6-phosphate isomerase (Δ*pgi*) diverts glucose metabolism through an NADP^+^-preferring Zwf resulting in the cofactor imbalance and negative effect on the growth (12). The deletion of Zwf in E. coli (Δ*zwf*) led to increased metabolic flux via isocitrate dehydrogenase and malic enzyme to fulfill the metabolic demands of NAD(P)H (13). However, in P. putida, where multiple copies of *zwf* are reported, the deletions of *zwf*(s) resulted in poor growth on glucose (14), indicating the importance of Zwf in the ED pathway-dependent bacteria. The *zwf* copies varied from one (e.g., P. doughuensis and P. vranovensis), two (e.g., P. fluorescens and P. aeruginosa), three (e.g., P. japonica, P. putida, and P. alkylphenolica), or greater than or equal to four copies (e.g., A. vinelandii and M. methanica). However, in most of them, only one of the Zwf isozymes has been functionally characterized. Among these kinetically characterized Zwfs, most of them were NADP^+^-preferring/specific while some are dual cofactor specific or NAD^+^-preferring (14–18) Additionally, few enzymes were also reported to have cooperativity for the substrate, G6P (15, 16, 19, 20). However, the information about kinetic properties, catalytic efficiencies, expression patterns, and significance of multiple isozymes from a single bacterial strain is scanty.

Pseudomonas bharatica CSV86^T^ metabolized glucose via an intracellular phosphorylative route and lacks oxidative routes (gluconate and 2-ketogluconate route) (Fig. 1). Strain CSV86^T^ displayed a unique carbon source utilization hierarchy, where aromatics and organic acids were preferred over simpler carbon sources, such as glucose and glycerol, while aromatics and organic acids are co-metabolized (21–23). Enzymes involved in the metabolism of glucose are suppressed in the presence of aromatics. Besides these unique properties, the strain displays various ecophysiological properties (like heavy metal resistance, plant growth-promoting traits, etc.) which makes it an ideal candidate for bioremediation (22). On glucose, the strain displayed poor growth with a prolonged lag phase. Genome analysis revealed the lack of genes responsible for oxidative routes and the presence of three *zwf* genes. Therefore, it is of significance to study their involvement in glucose metabolism, their arrangement with respect to other glucose metabolism/transport genes, and their molecular and kinetic properties. The present study attempted to understand the expression profiling and catalytic efficiencies of multiple Zwfs in the glucose metabolism in strain CSV86^T^. This will help to understand the underlying mechanism of slower glucose metabolism and unique carbon source utilization hierarchy.

## RESULTS AND DISCUSSION

### Genome analysis revealed the presence of three copies of *zwf* in P. bharatica CSV86^T^.

**FIG 2:**
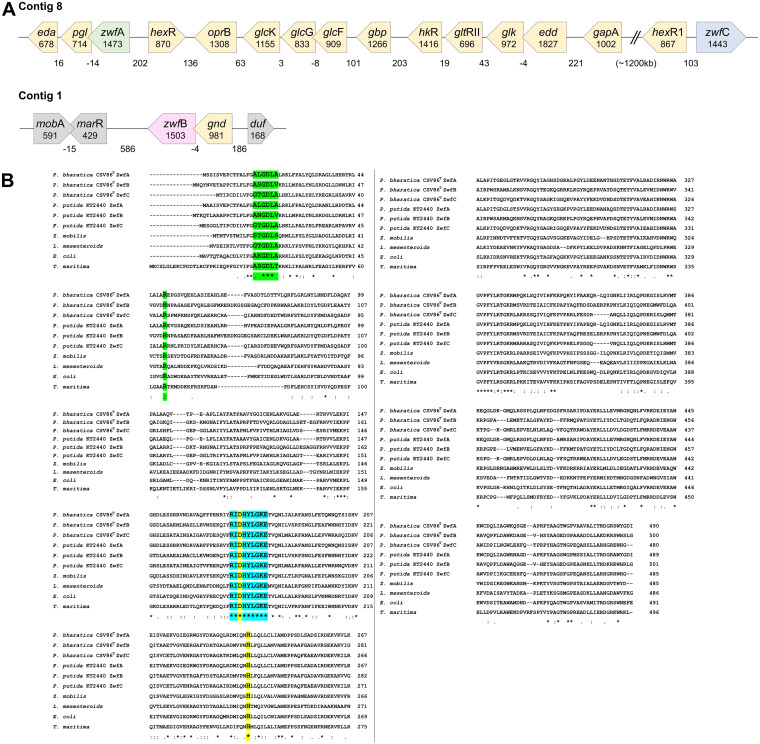
*In silico* analyses of glucose-6-phosphate dehydrogenases (Zwf) from Pseudomonas bharatica CSV86^T^. (A) Arrangement of *zwf* genes in the CSV86^T^ genome. Genes are depicted by pentagonal boxes labeled with names and its length (bp). The numbers below the boxes indicate intergenic spaces (bp). The figure is not to the scale. Gene annotations: contig 8 – *eda*, 2-keto-3-deoxy-6-phosphogluconate aldolase; *pgl*, 6-phosphogluconolactonase; *zwf*A, glucose-6-phosphate dehydrogenase; *hex*R, transcriptional regulator; *opr*B, carbohydrate-selective porin; *glc*K, ATPase component of glucose ABC transporter; *glc*G and *glc*F, transmembrane components of glucose ABC transporter; *gbp*, periplasmic glucose binding protein; *hk*R, two-component sensor histidine kinase; *glt*RII, response regulator; *glk*, glucokinase; *edd*, 6-phosphogluconate dehydratase; *gap*A, glyceraldehyde 3-phosphate dehydrogenase; *hex*RI, transcriptional regulator; *zwf*C, glucose-6-phosphate dehydrogenase. Contig 1, *mob*A, molybdenum cofactor guanylyl transferase; *mar*R, MarR family transcriptional regulator; *zwf*B, glucose-6-phosphate dehydrogenase; *gnd*, decarboxylating 6-phosphogluconate dehydrogenase; *duf*, DUF6026 family protein. (B) Multiple sequence alignment of Zwfs from P. bharatica CSV86^T^ with other structurally/functionally characterized Zwfs. Residues involved in the nucleotide-binding are highlighted in green; substrate binding in blue and catalytic dyad in yellow color.

The draft genome analysis reveals the presence of three *zwf* gene copies annotated as *zwf*A (1473 nt, 490 aa), *zwf*B (1503 nt, 500 aa), and *zwf*C (1443 nt, 480 aa) (Fig. 2A). *zwf*A and *zwf*C were located on the contig 8 (NCBI accession no. NZ_AMWJ02000002.1), while *zwf*B was present on the contig 1 (NCBI accession no. NZ_AMWJ02000001.1). The *zwf*A was found to be a part of the glucose metabolic cluster *zwf*A-*pgl*-*eda*, where *pgl* and *eda* encoded 6-phosphogluconolactonase and 2-keto-3-deoxy-6-phosphogluconate (KDPG) aldolase enzymes, respectively. The *hex*R, which encoded a putative transcriptional regulator of glucose metabolic genes was found to be located divergently upstream of *zwf*A. Genes involved in the glucose transport (*gbp*-*glc*F-*glc*G-*glc*K-*opr*B) (24, 25) and metabolism (*edd*-*glk*-*glt*RII-*hk*R) (26) were present as two independent transcription units (operons) upstream to the *zwf*A encompassing the total length of ~16 kb (Fig. 2A). The *zwf*C was found to be located ~1200 kb away from *zwf*A and next to *hex*R-like transcriptional regulator annotated as *hex*R1. The *zwf*B was found to be a part of a *gnd*-*zwf*B cluster where the *gnd* encoded the decarboxylating 6-phosphogluconate dehydrogenase enzyme involved in the PP pathway (Fig. 2A).

BLASTN/P and genome analysis were performed for Pseudomonas spp. harboring multiple *zwf* copies. In P. putida KT2440 (genome accession number NC_002947) and P. japonica NBRC (NZ_BBIR00000000), three copies of *zwf* were found to be present with similar gene arrangement as observed in strain CSV86^T^. In P. fluorescens Pf0-1 (NC_007492.2) and P. aeruginosa PAO1 (NC_002516.2), two copies of *zwf* were present, among which one of the *zwf* was associated with the glucose transport and metabolic genes, which was similar to *zwf*A of strain CSV86^T^. The other copy of *zwf* from P. fluorescens Pf0-1 showed a gene arrangement similar to *zwf*B, while in P. aeruginosa PAO1, the arrangement was found to be similar to *zwf*C of strain CSV86^T^. Apart from Pseudomonas spp., multiple *zwf* copies were also found to be present in most of the ED pathway-dependent microbes such as *Xanthomonas*, *Azotobacter*, *Methylomonas*, *Actinobacteria*, among others.

The pairwise amino acid sequence alignment of Zwf isozymes from strain CSV86^T^ showed ~40% to 50% identity among each other. The ZwfA, ZwfB, and ZwfC of strain CSV86^T^ shared 84.5, 82, and 84.1% identity with respective Zwf of P. putida KT2440, and 94.1%, 89.8%, and 95.6% identity with respective Zwf of P. japonica (unpublished data). The multiple amino acid sequence alignment of Zwf from CSV86^T^ with structurally and functionally characterized Zwf from Leuconostoc mesenteroids (27) and functionally characterized Zwfs from P. putida (14), Zymomonas mobilis (28), E. coli (18), and Thermotoga maritima (29) depicted in Fig. 2B revealed that (i) the conserved active site residues involved in the nucleotide binding, the (G/A)X_1-2_GXX(G/A) motif with Rossmann fold (βαβ) at N-terminal region, the arginine residue at β2-domain, and (ii) substrate binding, i.e., the nonapeptide motif RIDHYLGKE and the catalytic dyad (His240-Asp177) where His240 acted as the general base abstracting a proton from the C_1_-hydroxyl group of G6P and Asp177 helped in stabilizing the positive charge of His240 in the transition state (27, 30–32). Most of the reported Zwf preferred NADP^+^ as a cofactor and possessed a conserved arginine residue at β2-domain. This arginine residue formed four hydrogen bonds with the phosphate group of NADP^+^ (18, 27, 30, 33). In strain CSV86^T^, ZwfA, and ZwfC were found to possess conserved arginine residue at position 49 and 54 in the β2-domain, respectively, while ZwfB had histidine at corresponding position 52. Besides arginine in the β2-domain, the lysine residue in the glycine-rich β1α1-loop has been reported to be involved in the interaction with NADP^+^. The mutation of this lysine to alanine resulted in a reduced affinity to NADP^+^ (18). However, the lysine residue was not predominantly conserved across the bacterial species and was often found to be substituted with leucine, threonine, serine, or asparagine. In strain CSV86^T^, the residue found to be present at the position corresponding to β1α1-loop lysine was leucine (L17) in ZwfA, serine (S20) in ZwfB and threonine (T14) in ZwfC, which could alter the affinity of these isozymes for NAD(P)^+^.

**FIG 3:**
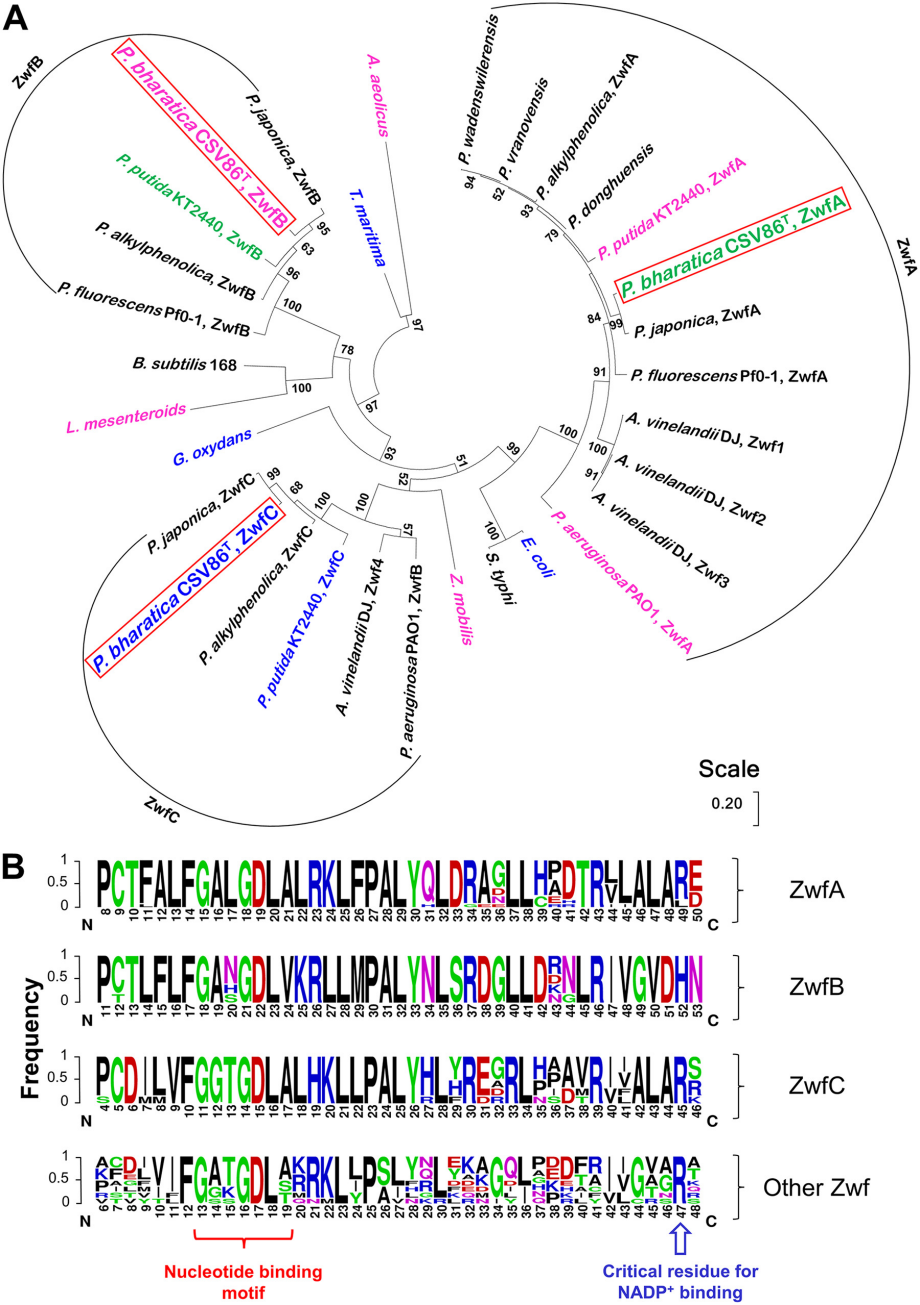
Phylogenetic analysis of glucose-6-phosphate dehydrogenases (Zwf) from Pseudomonas bharatica CSV86^T^. (A) Rooted phylogenetic tree depicting the distribution of Zwf isozymes into distinct clades. The functionally and kinetically characterized Zwfs were NADP^+^-specific in blue font, NADP^+^-preferring in pink font, and dual cofactor-specific in green font. Functionally uncharacterized Zwfs are in black font. Numbers at the nodes indicate the bootstrap values. (B) Consensus logo representing the conserved nucleotide binding motif and residues responsible for cofactor specificity in clades ZwfA, ZwfB, ZwfC, and other Zwf.

The rooted phylogenetic analysis revealed separate clades for ZwfA, ZwfB, and ZwfC, suggesting that three isoforms might share a distant common ancestor (Fig. 3A). Zwf from Pseudomonas members possessing single copy were observed to cluster with ZwfA. Exceptionally, out of four Zwfs in Azotobacter vinelandii DJ, three were found to cluster with ZwfA while the fourth Zwf with ZwfB. Zwf from non-Pseudomonas members were found to cluster in different clades. It was also observed that ZwfA and ZwfC were more closely related, while ZwfB displayed early diversion in the phylogenetic tree and, thus, distantly related to ZwfA and ZwfC. It was observed that the Zwf in each clade shared >69% amino acid sequence identity with the respective Zwf of strain CSV86^T^ (unpublished data). Such high amino acid sequence identities among Zwfs suggested probable similar functional features of these enzymes in the respective organism.

The key amino acid residues/motifs involved in the cofactor specificity were analyzed in each clade, revealing the presence of conserved nucleotide binding glycine-rich motif at β1α1 fold in all Zwfs. Interestingly, an anomaly was found at a position corresponding to the β2-arginine residue, which was majorly involved in NADP^+^ binding. All Zwfs except for the ZwfB group showed conservation of this arginine residue thus indicating a probable preference for NADP^+^, whereas ZwfB showed conservation of histidine residue at the corresponding position (Fig. 3B). Furthermore, to support these observations and clustering pattern, the cofactor preference of the functionally reported Zwfs was derived using the ratio of catalytic efficiency (*k_cat_*/*K_m_*) with NADP^+^ to NAD^+^. In the case where *k_cat_* was not determined, the ratio of affinity constant (*K_m_*) for NADP^+^ to NAD^+^ was used. Based on the comparison of kinetic properties, the Zwfs were classified into three categories: NADP^+^-specific if the ratio was >10-fold; NADP^+^-preferring if the ratio was 2 to 10-fold; and dual-cofactor specific if the ratio was in between 0.5 to 2-fold (Fig. 3A). It was observed that most of the Zwfs were NADP^+^-preferring or NADP^+^-specific, whereas only one Zwf have been reported among ZwfB (from P. putida KT2440), which was observed to display dual cofactor specificity.

### Cotranscription analysis revealed the operonic organization of *zwf* loci.

**FIG 4:**
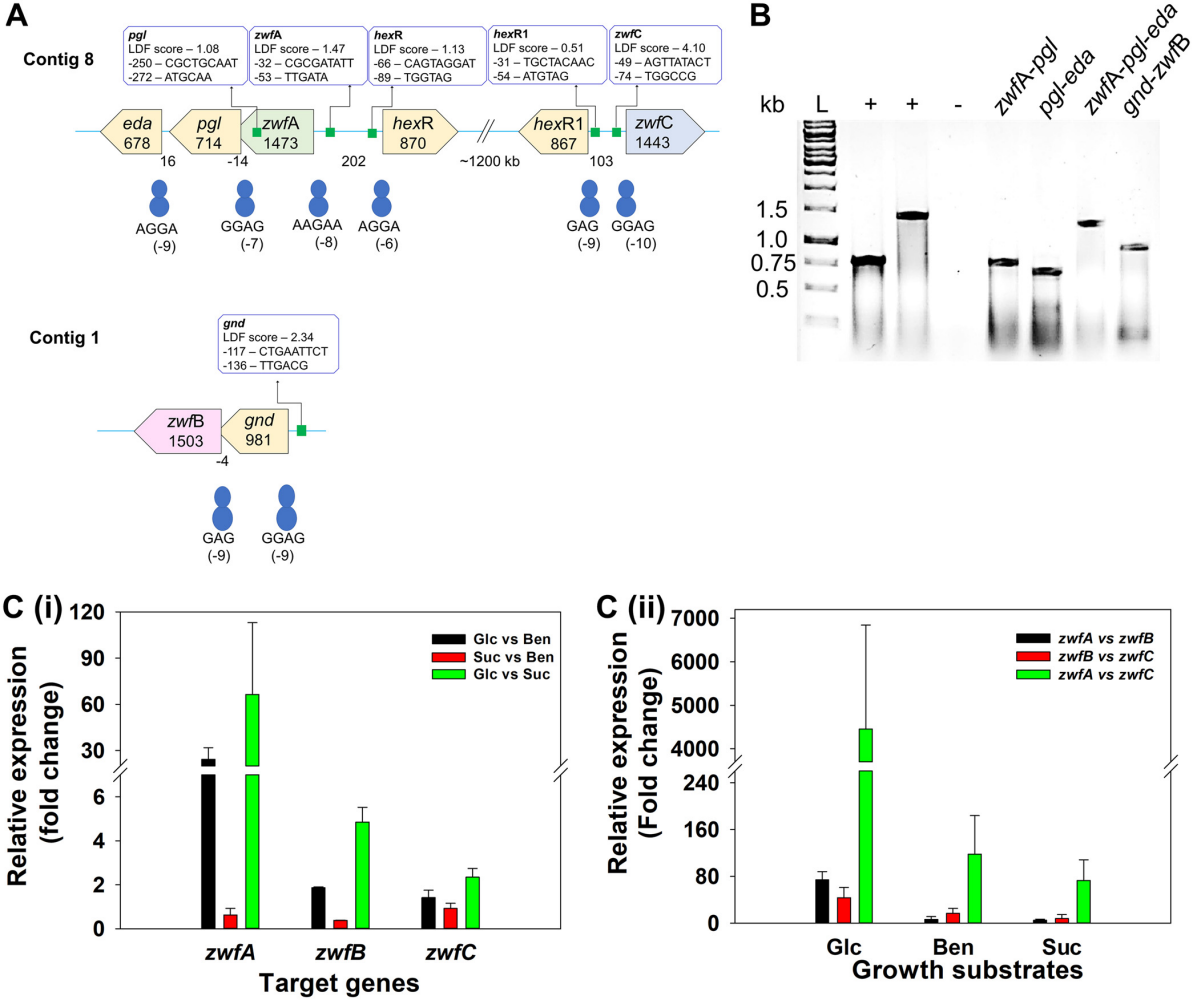
Cotranscription and qPCR analyses of *zwf* loci in Pseudomonas bharatica CSV86^T^. (A) Gene arrangement and promoter analysis of loci harboring *zwf*A, *zwf*B, and *zwf*C genes. Genes are depicted in the pentagonal boxes labeled with gene names and its length (bp). The numbers below boxes indicate the length of the intergenic region (bp). The green-filled squares indicate the predicted promoters and their details. The ribosome binding sites are indicated by blue circles with their predicted sequence and position. The figure is not to the scale. (B) Cotranscription analysis of *zwf*A and *zwf*B loci. Lane L, DNA ladder; lanes 2 and 3 (+), positive controls *crc* (expected length ~780bp) and *zwf*A (~1473 bp), respectively, using total DNA as the template; lane 4 (−), negative control (no cDNA template); lanes 5 to 8, PCR amplification using cDNA template and cotranscription primers for *zwf*A*-pgl* (~750 bp); *pgl-eda* (~650bp); *zwf*A*-pgl-eda* (~1350 bp); and *gnd-zwf*B (~900bp), respectively. (C) Carbon source-dependent comparison of the relative expression (fold change) of (i) *zwf* isozyme alone and (ii) pairwise comparison.

The gene arrangement, promoter prediction, and ribosome binding sites are depicted in Fig. 4A. In 5′-*zwf*A*-pgl-eda*-3′ cluster (*zwf*A locus), promoters were predicted upstream to *zwf*A and *pgl* genes with linear discriminant function (LDF) scores of 1.47 and 1.08, respectively. At the 5′-*gnd-zwf*B-3′ cluster (*zwf*B locus), a promoter upstream to *gnd* with an LDF score of 2.34 was detected, while the *zwf*C was found to be present independently possessing an upstream promoter with an LDF score of 4.10 (Fig. 4A). Based on gene arrangements, the cotranscription analyses were performed for *zwf*A and *zwf*B loci using specific intergenic primers (Table 1). At the *zwf*A locus, due to the presence of a weak *pgl* promoter, the forward primer designed for *zwf*A-*pgl* was upstream to the *pgl* promoter to demonstrate the polycistronic nature of mRNA for *zwf*A-*pgl-eda*. The electrophoretic analysis of various PCR products is depicted in Fig. 4B. The PCR products obtained using the intergenic primer pairs for *zwf*A*-pgl*, *pgl-eda*, *zwf*A-*pgl-eda*, and *gnd-zwf*B were ~750, 650, 1350, and 900 bp lengths, respectively. These amplicons corresponded to their expected length (Table 1), which were further gel purified and sequence confirmed (unpublished data). These results supported the gene arrangement at loci *zwf*A and *zwf*B and their cotranscription, suggesting transcription units (operons). Observed results were in accordance with previous reports for the *zwf*A locus from P. putida strains H and KT2440 (9, 34) and *zwf*B locus from P. fluorescence (35).

**TABLE 1:** List of primers used in the present study

Gene	Primer name^*a*^	Sequence (5′–3′)	Length of amplicon (bp)	Annealing temp. (°C)
Primers for cotranscription				
*zwf*A*-pgl*	zwfA_pgl_FP	TGATGACCAAGGAACAGGGC	752	60
	zwfA_pgl_RP	GGGAACAGCGAAGCGGTGT		
*pgl-eda*	pgl_eda_FP	TCTATCGCCACGCCGCCAGC	630	65
	pgl_eda_RP	TACCGGCGCCGATGCACAG		
*zwf*A-*pgl-eda*	zwfA_eda_FP	TGATGACCAAGGAACAGGGC	1382	60
	zwfA_eda_RP	TACCGGCGCCGATGCACAG		
*gnd-zwf*B	gnd_zwfB_FP	AGCGAGCTGAAAAAACGTCATCT	925	60
	gnd_zwfB_RP	GTCGCCGAGACGTTGAACCA		
Primers for gene-specific expression analysis using quantitative PCR				
*zwf*A	zwfA_qFP	AGGTCACCCCCGAAGCGCC	189	60
	zwfA_qRP	TCCGGGAAGAACTGCGCCAC		
*zwf*B	zwfB_qFP	GCAAGGGCGCAGGAAAGCTCAA	208	60
	zwfB_qRP	CGAAATTGCGCGTACGGTGC		
*zwf*C	zwfC_qFP	TTTTCAAAAGCTTGCAGAGCGC	185	60
	zwfC_qRP	GCCAGGTAATAGATCCGCGCCAG		
*rpo*D	rpoD_qFP	GACAGTGACGACGAAGACGA	155	60
	rpoD_qRP	TCACGACCATGCTTCTTGAG		
Primers for cloning in pET28a expression vector				
*zwf*A	zwfA_FP	CGGAATTCATGTCTTCGATCAGTGTTG	*Eco*RI	65
	zwfA_RP	TACTCGAGTCAGATATCGCCATACCAG	*Xho*I	
*zwf*B	zwfB_FP	ATCATATGATGAACCAGTACAACGTCG	*Nde*I	60
	zwfB_RP	ATAGTCGACTCAGCCCAGGCTATGC	*Sal*I	
*zwf*C	zwfC_FP	ATCATATGTTGACTATTCCTTGTGAC	*Nde*I	65
	zwfC_RP	TACTCGAGCTAGCCGTGCCATTCCCG	*Xho*I	

a FP, forward primer; RP, reverse primer; restriction enzyme sites are underlined.

### ZwfA was the key player in glucose metabolism.

P. bharatica CSV86^T^ prefers aromatic and organic acids over glucose by suppressing genes involved in glucose metabolism (21, 22). The specific activity of Zwf in the cell-free extract (CFE) was found to be ~5-fold higher in glucose-grown cells (450 ± 21 nmol min^−1^ mg^−1^) compared to naphthalene-grown cells (84 ± 8 nmol min^−1^ mg^−1^).

qPCR analyses revealed the maximum expression of *zwf* in the glucose-grown cells compared to benzoate or succinate (Fig. 4C). However, the relative expression of *zwf*A, *zwf*B, and *zwf*C was observed to be variable depending on the carbon sources used for the growth (Fig. 4C). In glucose-grown CSV86^T^ cells, the gene *zwf*A displayed ~35- or 72-fold higher expression, while *zwf*B showed ~3 or 5.5-fold, and *zwf*C 2- or 2.5-fold higher expression compared to benzoate or succinate grown cells, respectively (Fig. 4C). These results confirmed the inducible nature of glucose metabolic genes, and all three isoforms were found to display higher expression in glucose-grown CSV86^T^ cells. Furthermore, the *zwf*A expression in the glucose-grown cells was found to be ~75 and 4500-fold higher than *zwf*B and *zwf*C, respectively (Fig. 4C). The expression analyses suggested that ZwfA was the predominant isozyme involved in glucose metabolism in strain CSV86^T^. In P. putida KT2440, the deletion *of zwf*A showed poor growth in glucose, while the deletion of *zwf*B and/or *zwf*C had a negligible effect on the growth (14). qPCR analyses for *zwf* in strain CSV86^T^ and deletion mutant analyses in strain KT2440 corroborated the significant role of ZwfA in glucose metabolism.

In Pseudomonas, glucose metabolic genes are regulated by HexR, the transcriptional regulator (36, 37). It binds specifically to the conserved pseudopalindromic DNA sequences at the promoter region suppressing the target genes (38). In P. bharatica CSV86^T^, *hex*R was located divergently upstream to *zwf*A. Similarly, a *hex*R-like gene annotated as *hex*R1 was found to be present divergently upstream to *zwf*C (Fig. 2A). HexR and HexR1 shared ~62% amino acid sequence similarity. Furthermore, the sequence alignment of the consensus binding regions and its comparison with P. putida KT2440 revealed the presence of HexR binding sites each for *zwf*A and *zwf*C genes but were absent for *zwf*B (unpublished data). Interestingly, the gene *zwf*B was found to be a part of the transcription unit with *gnd* (the PP pathway gene), indicating its exclusion from HexR binding and regulation. This might aid in fine-tuning metabolic processes to meet the cellular requirement of ATP, NAD(P)H, or pentose sugars under glucose repression conditions.

### Kinetic properties supported ZwfA as the key enzyme.

Three isozymes were cloned into the pET vector (pET28a-*zwf*A, pET28a-*zwf*B, and pET28a-*zwf*C) and overexpressed individually into E. coli BL21(DE3). The CFE prepared from optimally-induced E. coli cells showed the specific activity of ~18.5 μmoles min^−1^ mg^−1^ for ZwfA, which was ~8 and 23-fold higher than that observed for ZwfB (~2.4 μmoles min^−1^ mg^−1^) and ZwfC (~0.8 μmoles min^−1^ mg^−1^), respectively. ZwfA and ZwfB utilized both NADP^+^ and NAD^+^ as cofactors while ZwfC utilized only NADP^+^ as a cofactor and displayed negligible enzyme activity in the presence of NAD^+^. The activity of ZwfA was found to be stable (100%) compared to ZwfB (50% loss) and ZwfC (20% loss) in 48 h at 5°C in the CFE.

### Purification and kinetic properties of Zwf isozymes.

**FIG 5:**
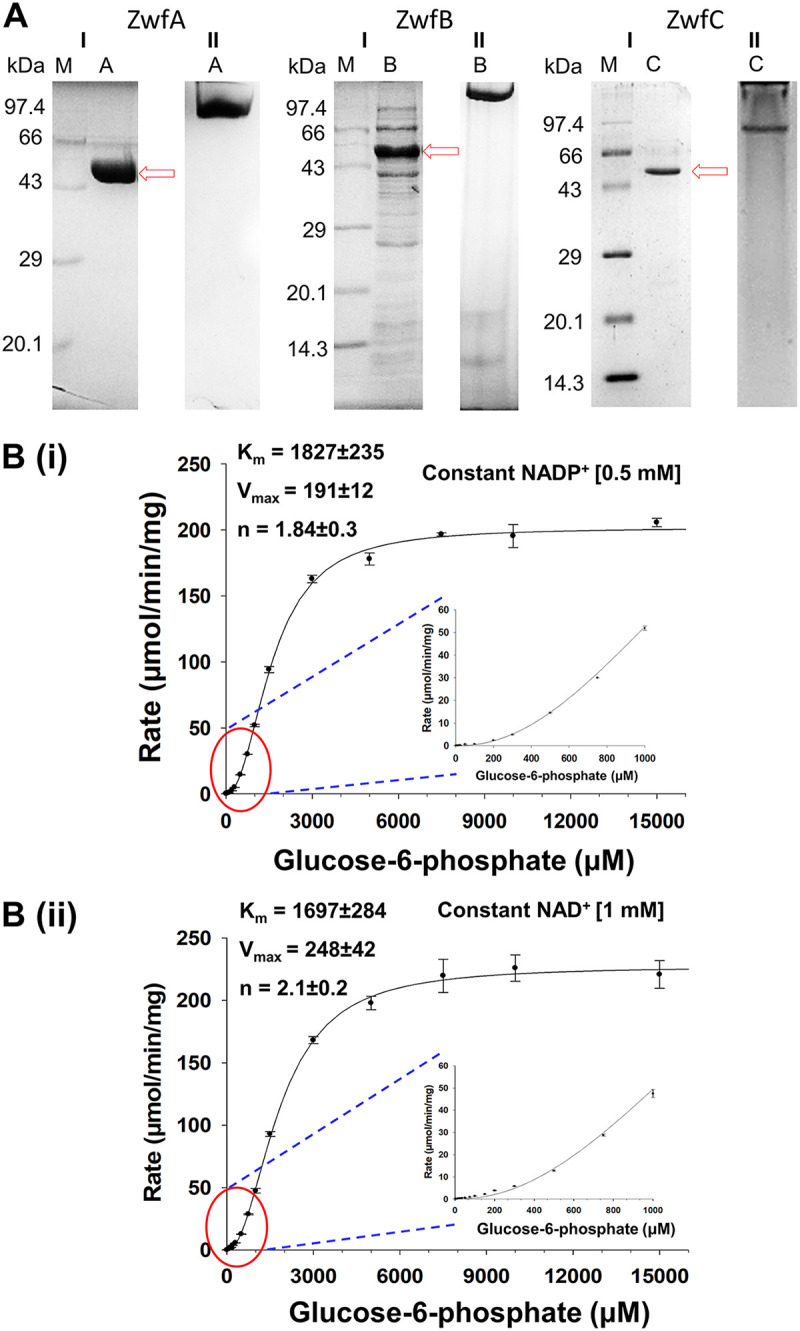
Purification of Zwf isozymes and kinetic properties of ZwfA from Pseudomonas bharatica CSV86^T^. (A) Electrophoretic mobility of the purified preparations of Zwf isozymes on (I) SDS-PAGE and (II) Native-PAGE; lane M represents molecular weight markers (kDa); lanes A, B, and C represent ZwfA, ZwfB, and ZwfC proteins, respectively, obtained after purification and marked by a red color arrow. (B) Sigmoidal substrate saturation profile of ZwfA in the presence of fixed concentration of cofactor (i) NADP^+^ and (ii) NAD^+^. Plots were constructed using Hill’s equation. The sigmoid region marked by a red circle is enlarged and shown as an inset plot for better clarity.

The overexpressed ZwfA, ZwfB, and ZwfC isozymes were purified from optimally induced E. coli using NiNTA affinity chromatography. The concentration range for imidazole to elute bound isoenzymes was 180 to 250 mM (for ZwfA), 100 to 175 mM (ZwfB), and 50 to 120 mM (ZwfC). ZwfA and ZwfC were obtained in the pure form while ZwfB retained impurities in the preparation (Fig. 5A). ZwfA was purified to 4.1-fold, 71% yield and specific activity of 76.4 μmoles min^−1^ mg^−1^, while ZwfC was purified to 9.9-fold, 21% yield and specific activity of 7.6 μmoles min^−1^ mg^−1^ using NiNTA column chromatography. From SDS-PAGE analyses, the subunit molecular weights were found to be ~56, 52, and 57 kDa for ZwfA, ZwfB, and ZwfC, respectively. The native molecular weight was determined by monitoring the elution profiles of standard molecular weight proteins on size exclusion chromatography. The elution profile for purified ZwfA was monitored using Enrich SEC650 column (CV = 24 mL, Bio-Rad), and for purified ZwfC using Superdex 300 increase 10/300GL column (CV = 24 mL, GE Healthcare [Cytiva]). From the semilog plot of *V_e_*/*V_0_* (elution/void volume) on the *x*-axis and log_10_(molecular weight) on the *y*-axis, the native molecular weight of ZwfA and ZwfC were determined to be ~217 and 207 kDa, respectively, suggesting them to be homotetrameric. Due to impurities present in ZwfB preparation, the native molecular weight could not be determined. The reported quaternary structures of Zwf vary from homodimer, tetramer, or octamer. In L. mesenteroids, Gluconobacter oxydans, Aquifex aeolicus, Burkholderia licheniforms, T. maritima, and B. cepacian, Zwfs were reported to be homodimeric (29, 39–42). While, in Z. mobilis and A. vinelandii, Zwfs were reported to be homotetrameric (16, 28). In P. aeruginosa, the Zwf was reported to be present in two states, homotetramer and homooctamer (20).

ZwfA and ZwfC showed optimal activity in Tris-Cl buffer, 50 mM, pH 8.25 while ZwfB showed optimal activity in citrate-phosphate buffer 50 mM pH 6.5. At fixed concentration, 4 mM G6P and 200 μM NADP^+^ or NAD^+^, the specific activity (μmoles min^−1^ mg^−1^) of ZwfA was 128 ± 11 with NADP^+^ and 140 ± 26 with NAD^+^. For ZwfB, it was 3.6 ± 3.5 with NADP^+^ and 1.64 ± 1.4 with NAD^+^. For ZwfC, it was 8.4 ± 1.5 with NADP^+^ and 0.61 ± 0.2 with NAD^+^. The kinetic constants were determined at the optimum pH and are summarized in Table 2. ZwfA showed a sigmoidal substrate saturation profile for G6P in the presence of NADP^+^ (Hill’s coefficient, *n* = ~1.84, Fig. 5B) as well as with NAD^+^ (Hill’s coefficient, *n* = ~2.1, Fig. 5B). ZwfB and ZwfC displayed hyperbolic substrate saturation profile for G6P in the presence of either NADP^+^ or NAD^+^ (unpublished data). At a fixed concentration of G6P, all isozymes displayed hyperbolic substrate saturation profiles for NADP^+^ or NAD^+^ (unpublished data). The ZwfA showed an equal affinity for G6P irrespective of NADP^+^ or NAD^+^ but at a fixed concentration of G6P, it showed ~3-fold higher affinity for NADP^+^ (Table 2). The ZwfB displayed ~2-fold higher affinity for G6P in the presence of NADP^+^ and ~9-fold higher affinity toward NADP^+^ at a fixed concentration of G6P (Table 2). While ZwfC showed ~5-fold higher affinity for G6P in the presence of NAD^+^ and ~175-fold higher affinity toward NADP^+^ at the fixed concentration of G6P (Table 2). The catalytic efficiency (*k_cat_*/*K_m_*) was ~2 and ~120-fold higher for NADP^+^ for ZwfA and ZwfC, respectively (Table 2). The ZwfA appeared to be catalytically more efficient (higher *k_cat_*/*K_m_*) compared to ZwfC (Table 2). These results suggested that ZwfA was dual cofactor specific, ZwfB was NADP^+^-preferring, and ZwfC was an NADP^+^-specific isozyme. The majority of the Zwf displayed a preference for NADP^+^, although few dual cofactor-specific or NAD^+^-preferring were reported (14–18). Notably, the dual cofactor specificity of Zwf was linked to the presence of its multiple isoforms, where this feature was observed in at least one-of-the isoforms. The presence of multiple *zwf* genes and, thus, probable dual cofactor utilization characteristic was found majorly in ED pathway-preferring organisms. The higher catalytic efficiency of ZwfA with positive cooperativity (*n* = 2, allosteric property) further supported it to be a key enzyme involved in the metabolism of glucose under dynamic substrate concentration conditions. The dual-cofactor specificity of ZwfA might further aid in conserving the intracellular NAD(P)^+^/NAD(P)H ratios, and its allosteric nature assisted in regulating the utilization of glucose-6-phosphate. Such fine-tuning of metabolic processes may help the organism to attain physiological robustness and ultimately yield survival benefits. Such allosteric property has been previously reported in P. aeruginosa, A. vinelandii, and P. fluorescens with ‘n’ ranging from 1.6 to 2.4 (15, 16, 20).

**TABLE 2:** Kinetic constants of glucose-6-phosphate dehydrogenases (ZwfA, ZwfB, and ZwfC) from Pseudomonas bharatica strain CSV86^T^

Enzyme	G6P with fixed NADP^+^ conc.					G6P with fixed NAD^+^ conc.					NADP^+^ with fixed G6P conc.				NAD^+^ with fixed G6P conc.			
	*K_m_* ^*a*^	*V_max_*	*K_cat_*	*k_cat_/K_m_*	*n*	*K_m_*	*V_max_*	*k_cat_*	*k_cat_/K_m_*	*n*	*K_m_*	*V_max_*	*K_cat_*	*k_cat_/K_m_*	*K_m_*	*V_max_*	*K_cat_*	*k_cat_/K_m_*
ZwfA^*b*^	1827 ± 235	191 ± 12	177 ± 11	0.096 ± 0.016	1.84 ± 0.3	1697 ± 284	248 ± 42	230 ± 37	0.16 ± 0.04	2.1 ± 0.17	110.5 ± 20	201 ± 28	187 ± 26	1.7 ± 0.3	366 ± 70	329 ± 74	306 ± 69	0.86 ± 0.15
ZwfB^*c*^	5827 ± 94	ND^*d*^	ND	ND	NA^*e*^	9968 ± 2716	ND	ND	ND	NA	67.5 ± 3	ND	ND	ND	577 ± 34	ND	ND	ND
ZwfC	1940 ± 172	11.4 ± 2.7	10.9 ± 2.6	0.0056 ± 0.0014	NA	364 ± 22	11.9 ± 1.6	11.4 ± 1.6	0.03 ± 0.0043	NA	18 ± 4.4	8.9 ± 1.6	8.5 ± 1.5	0.48 ± 0.05	3100 ± 285	12.4 ± 3.3	11.9 ± 3.2	0.0041 ± 0.0014

a *K_m_*, μM; *V_max_*, μmol min^−1^ mg^−1^; *k_cat_*, s^−1^; *k_cat_/K_m_*, μM^−1^ s^−1^.

b Enzyme displayed cooperativity for G6P binding (see Fig. 5B).

c Kinetics were performed using a partially purified enzyme.

d ND, not determined due to impure ZwfB preparations.

e NA, not applicable because enzyme displayed a hyperbolic Michaelis-Menten saturation profile under these conditions.

In strain CSV86^T^, isozymes displayed different kinetic properties and cofactor utilization patterns. ZwfA with dual cofactor specificity and cooperativity played a major role, while ZwfB and ZwfC had a minor role in glucose metabolism. ZwfB probably participated in the PP pathway and ZwfC with low catalytic efficiency potentially evolved to function differently. ZwfA displayed ~2- to 120-fold lower affinity for G6P (higher *K_m_*), as well as significantly lower catalytic efficiency, compared to Zwfs, reported from other organisms (Table 3). The strain displayed a lower specific growth rate on glucose (0.24 h^−1^, a prolonged lag phase of ~10 h and stationary phase at ~20 h) compared to organic acids (~0.6 h^−1^ and a lag phase of ~2 h for succinate, fumarate, pyruvate, or α-ketoglutarate) and aromatics (~0.55 h^−1^ and a ~2 h lag phase for naphthalene or benzoate) (21–23). The absence of the oxidative routes and lower catalytic efficiency of Zwf were probable reasons for the slower growth of strain CSV86^T^ on glucose. In P. putida KT2440, which has intracellular phosphorylative as well as both oxidative routes for glucose metabolism, displayed faster growth on glucose (0.25%) with a specific growth rate of ~0.56 h^−1^ (growth profiles are unpublished data).

**TABLE 3:** Comparison of kinetic properties of glucose-6-phosphate dehydrogenases

Organism	G6P with fixed conc. of NADP^+^				G6P with fixed conc. of NAD^+^				NADP^+^ with fixed conc. of G6P			NAD^+^ with fixed conc. of G6P			*k_cat_*/*K_m_*(NADP^+^)/ *k_cat_*/*K_m_*(NAD^+^)	Quarternary structure	Subunit mol. wt. (kDa)	pH optima	References
	*K_m_* ^*a*^	*k_cat_*	*k_cat_*/*K_m_*	SS^*b*^	*K_m_*	*k_cat_*	*k_cat_*/*K_m_*	SS^*b*^	*K_m_*	*k_cat_*	*k_cat_*/*K_m_*	*K_m_*	*k_cat_*	*k_cat_*/*K_m_*					
P. bharatica CSV86^T^ (A)^*c*^	1827	191	0.10	S (1.84)	1697	230	0.16	S (2.1)	110	187	1.7	366	306	0.86	2	Homotetramer	55.8	8.25	Current study
P. bharatica CSV86^T^ (B)	5827	NA^*d*^	NA	M	9968	NA	NA	M	67	NA	NA	577	NA	NA	NA	NA	51.5	6.5	
P. bharatica CSV86^T^ (C)	1940	11.4	0.0056	M	364	11.4	0.03	M	18	8.5	0.48	3100	11.9	0.004	123	Homotetramer	57.5	8.25	
T. maritima	200	35000	175	M	NA	NA	NA	NA	40	35000	875	12000	11000	0.92	955	Homodimer	60	7.4	29
E. coli	NA	NA	NA	NA	NA	NA	NA	NA	7.5	174	23.2	5090	288	0.06	410	NA	NA	8.2	18
G. oxydans	280	44	0.16	M	NA	NA	NA	NA	26	43	1.6	740	43	0.06	28	Homodimer	54.5	7	42
L. mesenteroids	114	523	4.6	M	69	1125	16.3	M	8	522	65.3	162	1125	6.9	9.4	Homodimer	-	7.6	39
A. aeolicus 40°C	15	NA	NA	M	58	NA	NA	M	9.1	47	5.1	230	58	0.25	20	Homodimer	55	7	41
A. aeolicus 70°C	63	NA	NA	M	180	NA	NA	M	161	894	5.6	2096	2012	0.96	5.8				
Z. mobilis	NA	NA	NA	M	NA	NA	NA	M	40	338	8.5	210	589	2.8	3	Homotetramer	52	8	28
P. fluorescens	2700	NA	NA	S (1.65)	2330	NA	NA	S (1.59)	360	1117	3.1	150	1383	9.2	0.34	NA	NA	8.9	15
P. putida KT2440 (A)	946	NA	NA	M	1137	NA	NA	M	14	102	7.3	127	227	1.8	4.1	NA	NA	8	8
P. putida KT2440 (B)	190	NA	NA	M	291	NA	NA	M	165	113	0.68	151	120	0.8	0.86	NA	NA	8	14
P. putida KT2440 (C)	944	NA	NA	M	2030	NA	NA	M	3.2	0.54	0.17	9500	0.77	8.1 × 10^−5^	2082	NA	NA	8	
P. aeruginosa (A)	499	NA	NA	S (1.99)	1146	NA	NA	S (2.38)	57	540	9.5	527	1017	1.9	4.9	Homotetramer or octamer	55	8	20
A. vinelandii	530	NA	NA	S (1.66)	690	NA	NA	S (1.76)	50	36.7	0.73	220	91	0.41	1.8	Homotetramer	52	8.5	16

a *K_m_*, μM; *k_cat_*, s^−1^; *k_cat_/K_m_*, μM^−1^ s^−1^.

b SS, substrate saturation profile: S, sigmoidal saturation profile with Hill’s coefficient (n) value in the bracket indicating the allosteric nature of enzyme; M, Michaelis-Menten hyperbolic saturation profile.

c Zwf A, B, or C isozymes.

d NA, data not available.

The presence of multiple genes might be the result of segmental duplication (paralogs) or horizontal gene transfer events (homologs). The gene duplication is either the cause (Ohno model) or consequence (IAD model) of the promiscuous side function (43). In E. coli, the divergence of three isozymes of 3-deoxy-7-phosphoheptulonate synthase after duplication allows the acquisition of differential feedback inhibition. Where each isozyme is inhibited by one of the aromatic amino acids thus controlling the flux into each pathway (43). In E. coli, a point mutation of Glu383 to Ala in l-gamma-glutamyl phosphate reductase (ProA, enzyme involved in proline synthesis) increased its promiscuous ability to participate in arginine biosynthesis by catalyzing the reduction of *N*-acetyl-l-gamma-glutamyl phosphate while decreasing its original activity as ProA (44). In eukaryotes, differential cellular localization of two NADP^+^-dependent isocitrate dehydrogenase isozymes has been reported where one is localized in both cytoplasm and peroxisome while the other is found in mitochondria. The NADPH-produced aids in different functions based on the location (45, 46).

Pseudomonas bharatica CSV86^T^ metabolized glucose solely via the intracellular phosphorylative ED route but with a low growth rate. The enzyme G6PDH (Zwf) was one of the key enzymes to generate redox cofactors, NAD(P)H during glucose metabolism. Genome analysis revealed the presence of three copies of *zwf* with 40% to 50% amino acid sequence identity and clustering into distinct phylogenetic clades. *zwf*A and *zwf*B were organized as individual transcription units as *zwf*A-*pgl*-*eda* and *gnd*-*zwf*B, respectively, whereas *zwf*C was present independently. The *zwf*B was found to be devoid of the consensus sequence for binding of transcription repressor HexR, indicating its probable role in the PP pathway. *zwf*A was expressed abundantly compared to *zwf*B and *zwf*C in CSV86^T^. All three isozymes were purified and kinetically characterized. Among them, ZwfA displays allosteric nature for substrate binding and higher catalytic efficiency. However, the catalytic efficiency was significantly low compared to other reported Zwfs. With respect to cofactor utilization, ZwfA exhibited dual-cofactor specificity, ZwfB displayed a preference for NADP^+^ while ZwfC was NADP^+^ specific. The substitution of key amino acids lysine by leucine (ZwfA), serine (ZwfB), or threonine (ZwfC) at β1α1-loop glycine-rich motif and replacement of arginine with histidine at the β2-domain in ZwfB might be responsible for the observed altered cofactor specificity. The varied cofactor preferences and allosteric nature might enable balance in glucose utilization and cofactor production. However, the low catalytic efficiency of Zwfs and lack of oxidative routes might lead to limiting concentration of downstream pathway intermediates, such as KDPG (one of the proposed modulators of the metabolic pathway through HexR (36)), thus affecting the growth on glucose. Such defective glucose metabolism might enable strain CSV86^T^ to preferentially utilize substrates, such as aromatics. The unique hierarchy of carbon sources hence can be further explored for its application in the field of bioremediation of toxic aromatic pollutants and aids in environmental cleanup.

## MATERIALS AND METHODS

### Microorganisms, growth, and culture conditions.

Pseudomonas bharatica CSV86^T^, Pseudomonas putida KT2440, and Escherichia coli strain DH5α and BL21(DE3) were used in the present study. Strain CSV86^T^ was grown on 150 mL minimal salt medium (MSM) pH 7.5 (47) supplemented aseptically (wt./vol.) with naphthalene (0.1%), benzoate (0.1%), succinate (0.25%), or glucose (0.25%) in 500 mL baffled Erlenmeyer flasks at 30°C on a rotary shaker (200 rpm). The E. coli strains DH5α and BL21(DE3) (Novagen) were grown on Luria-Bertani broth (LB) at 37°C on a rotary shaker at 200 rpm (48). Growth experiments for strains CSV86^T^ and KT2440 were performed on MSM medium supplemented with 0.25% glucose by measuring optical density at 540 nm using a spectrophotometer every 2 h.

### Genome mining for glucose metabolic genes and bioinformatic analyses.

The advanced version of the genome sequence of Pseudomonas bharatica CSV86^T^ was retrieved from NCBI (accession no. AMWJ02000000) (49). The nucleotide and amino acid sequences of glucose metabolic genes were extracted using BLASTN/P. The amino acid sequences of Zwf isozymes were retrieved from NCBI and PDB databases. Multiple sequence alignment was performed using Clustal Omega (https://www.ebi.ac.uk/Tools/msa/clustalo/) and phylogeny was constructed using MEGA 7.0 (50). The consensus signature sequence/logo was generated using the Weblogo tool (https://weblogo.berkeley.edu/logo.cgi). Promoters were predicted using the BPROM tool (http://www.softberry.com/) and scored as linear discriminant function (LDF) values. Ribosome binding sites were predicted/identified as described in (51).

### Molecular biology techniques.

**(i) Isolation of total RNA and cDNA synthesis.** Strain CSV86^T^ cells grown on glucose, benzoate, or succinate were harvested at the early log phase (optical density at 540 nm [OD_540_] = ~0.4) and total RNA was isolated using RNeasy Minikit (Qiagen, Germany) and made DNA-free using Turbo DNA-free kit (Invitrogen, USA) as described. The purity of DNA-free RNA was determined by A_260_/A_280_. cDNA synthesis was performed using DNA-free RNA as a template using the SuperScript-III First Strand cDNA synthesis kit (Thermofisher, USA).

**(ii) Cotranscription analysis.** Cotranscription analysis was carried out by performing PCR using cDNA prepared from CSV86^T^ cells grown on glucose as a template, appropriate primers (Table 1), Phusion DNA polymerase (NEB), and thermal cycler (Applied Biosystems, Thermofisher, USA). The PCR products were visualized on agarose gel (1%), eluted using an Expin Gel SV kit (GeneAll, South Korea), and sequence confirmed (1st base, Malaysia).

**(iii) Gene-specific expression profiling.** The quantitative PCR (qPCR) was performed using cDNA as a template (from glucose, benzoate, or succinate-grown CSV86^T^ cells), gene-specific primers (Table 1), and Platinum SYBR green qPCR SuperMix-UDG with ROX (Invitrogen, Thermo, USA). Signals were recorded using the StepOnePlus real-time PCR system (Applied Biosystems, Thermo, USA). Transcription of *rpo*D, the housekeeping gene was used as an internal control (52). Amplification of the target gene was analyzed based on the C_t_ value. The efficiency (*E*) of qPCR for one cycle in the exponential-phase was determined by *E *= 10^−1/slope^ (53). The fold change in expression was determined by the mathematical equation 2^−ΔΔCt^ (54).

### Cloning, expression of Zwf isozymes, and enzyme assay.

Genes *zwf*A, *zwf*B and *zwf*C were PCR amplified using total DNA from strain CSV86^T^ (isolated using Exgene Cell SV kit, GeneAll, S. Korea) as a template, Phusion polymerase and appropriate primers (Table 1). PCR products were gel purified, digested with appropriate restriction enzymes, and cloned into pET28a(+) expression vector with 6×His tag at the N-terminal (55). Recombinants were selected on LA+Kanamycin (Kan, 40 μg/mL) plates and confirmed by colony PCR and sequencing (1st base, Malaysia). The constructs were named as pET28a-*zwf*A, pET28a-*zwf*B and pET28a-*zwf*C.

E. coli BL21(DE3) cells were transformed with respective construct. A single colony was transferred into LB+Kan medium (5 mL) and grown overnight at 37°C on a rotary shaker (200 rpm). This primary culture was inoculated (1% vol/vol) into LB+Kan (100 mL) and grown until OD_540_ = 0.6 to 0.8 at 37°C followed by incubation on ice for 1 h. Cultures were induced at 25°C with isopropyl β-D-1-thiogalactopyranoside (IPTG): 100 μM for 6 h (for ZwfA) or 25 μM for 8 h (for ZwfB or ZwfC) on a rotary shaker. After induction, cells were harvested and washed twice with Buffer A (Tris-Cl buffer 50 mM, pH 7.5) for ZwfA and ZwfC, or Buffer B (Tris-Cl buffer, 50 mM pH 7.2 + NaCl, 100 mM + glycerol, 2% + dithiothreitol, 1 mM) for ZwfB. Cells (1 g) were resuspended in the respective buffer (5 mL, ice-cold) and lysed using an ultrasonic processor (GE 130, Cole-Parmer, USA) with 8 cycles of 25 pulses each (1 s pulse, 1 s interval, cycle duration 50 s, output 11 to 14 W and 5 min intervals between cycles) on ice. The cell-free extract (CFE) was prepared by successive centrifugation of cell homogenate at 30000 × *g* for 30 min followed by ultracentrifugation (Beckman Coulter, USA) at 100,000 × *g* for 1 h at 5°C. Protein concentration was estimated as described in (56) using bovine serum albumin (BSA) as the standard.

Glucose-6-phosphate dehydrogenase (Zwf) activity was monitored spectrophotometrically (Lambda35, PerkinElmer) by measuring the rate of increase in the absorbance due to the appearance of NAD(P)H at 340 nm (ɛ_340nm_ = 6220 M^−1^ cm^−1^) (21). The reaction mixture (1 mL) contained glucose-6-phosphate (4 mM), NADP^+^ or NAD^+^ (200 μM), an appropriate amount of CFE and buffer (Tris-Cl 50 mM, pH 8.25 for ZwfA and ZwfC, or Citrate phosphate 50 mM, pH 6.5 for ZwfB).

### Protein purification.

All protein purification steps were performed at 4°C.

For the purification of ZwfA, the protein was purified using FPLC (NGC quest plus, Bio-Rad, USA) at a constant flow rate of 30 mL h^−1^. The CFE prepared from E. coli cells overexpressing ZwfA was loaded on to Ni-NTA affinity chromatography matrix (~5mg/mL of matrix, column volume, CV, 5 mL, Qiagen, Germany) pre-equilibrated with Buffer A. The column was washed with Buffer A (4 × CV) followed by 2 × CV each of 50 and 100 mM imidazole solution in Buffer A. Bound ZwfA was eluted using a linear gradient of imidazole (100 to 350 mM, 5 × CV) in Buffer A. The active and pure fractions were pooled, concentrated using 30K centricon (PALL Corporation, USA) and chromatogramed on the size exclusion chromatography (CV = 24 mL, ENrich SEC 650 column, Bio-Rad, pre-equilibration and developed with Buffer A).

For the purification of ZwfB, the CFE (~ 5 mg protein/mL matrix) prepared from E. coli cells overexpressing ZwfB was loaded onto Ni-NTA affinity chromatography matrix (5 mL, Qiagen, Germany) pre-equilibrated with Buffer B. The column was washed with Buffer B (4 × CV) and bound protein was eluted using a linear gradient of imidazole (0 to 300 mM, 10× CV) in Buffer B. Active fractions were pooled and concentrated using centricon (30K) and chromatogramed on the size exclusion chromatography (CV = 24ml, ENrich SEC 650 column, Bio-Rad, pre-equilibration and developed with Buffer B).

For the purification of ZwfC, the protein was purified by using the protocol as described for ZwfB using Buffer A. Active and pure fractions were pooled and concentrated using centricon (30K). The native molecular weight was determined by size exclusion chromatography (CV = 24 mL), Superdex 200 Increase 10/300 GL column (GE Healthcare [Cytiva], USA) pre-equilibrated and developed with Buffer A.

To determine the native molecular weight by size exclusion chromatography the molecular weight markers used were: cytochrome c (12.4 kDa), carbonic anhydrase (29 kDa), bovine serum albumin (66 kDa), alcohol dehydrogenase (150 kDa), β-amylase (200 kDa), apoferritin (443 kDa), and thyroglobulin (669 kDa).

The purity of enzyme preparations was monitored using SDS-PAGE and Native-PAGE (57, 58). Subunit molecular weight was determined by SDS-PAGE using molecular weight markers: lysozyme (14.3 kDa), soybean trypsin inhibitor (20.1 kDa), carbonic anhydrase (29 kDa), ovalbumin (43 kDa), bovine serum albumin (66 kDa), and phosphorylase b (97.4 kDa).

### Kinetic characterization.

For ZwfA, the kinetic constants were determined using 1.3 μg of purified ZwfA per assay in Tris-Cl buffer (50 mM, pH 8.25), spectrophotometrically. The substrate saturation profiles were generated using (i) various concentrations of substrate, G6P (5 to 15000 μM) and fixed concentration of cofactor NADP^+^ (0.5 mM) or NAD^+^ (1 mM); and (ii) fixed concentration of G6P (5 mM) and various concentrations of NADP^+^ (2.5 to 1500 μM) or NAD^+^ (1 to 2000 μM).

For ZwfB, to determine the kinetic constants, 5 μg of partially purified ZwfB was used per enzyme assay in citrate-phosphate buffer (50 mM, pH 6.5), spectrophotometrically. The substrate saturation profiles were generated using (i) various concentrations of G6P (50 to 2 × 10^5^ μM) and fixed concentration of NADP^+^ (0.5 mM) or NAD^+^ (6 mM); and (ii) fixed concentration of G6P (5 mM) and various concentrations of NADP^+^ (2 to 5000 μM) or NAD^+^ (5 to 7500 μM).

For ZwfC, the kinetic constants were determined using 5 μg of purified ZwfC per assay in Tris-Cl buffer (50 mM, pH 8.25), spectrophotometrically. The substrate saturation profiles were generated using (i) various concentrations of G6P (20 to 5 × 10^4^ μM) and fixed concentration of NADP^+^ (0.25 mM), and various concentrations of G6P (20 to 10^4^ μM) and fixed concentration of NAD^+^ (15 mM); and (ii) fixed concentration of G6P (5 mM) and various concentrations of NADP^+^ (0.5 to 1000 μM) or NAD^+^ (50 to 3 × 10^4^ μM).

The kinetic constants and Hill’s coefficient (n) were determined by plotting specific activity data using SigmaPlot software (version 12) as Michaelis-Menten plot and Hill’s plot, respectively. Each experiment was performed at least in triplicates and each reading was in triplicates.

## References

[1] Koonin EV, Galperin MY. 2003. Chapter 7: evolution of central metabolic pathways: the playground of non-orthologous gene displacement, p 295–355. *In* Sequence - Evolution - Function: Computational Approaches in Comparative Genomics. Kluwer Academic Publishers, Boston. 21089240

[2] Nikel PI, Chavarría M, Fuhrer T, Sauer U, de Lorenzo V. 2015. *Pseudomonas putida* KT2440 strain metabolizes glucose through a cycle formed by enzymes of the Entner-Doudoroff, Embden-Meyerhof-Parnas, and Pentose Phosphate pathways. J Biol Chem 290:25920–25932. doi:10.1074/jbc.M115.687749. 26350459 PMC4646247

[3] Flamholz A, Noor E, Bar-Even A, Liebermeister W, Milo R. 2013. Glycolytic strategy as a trade-off between energy yield and protein cost. Proc Natl Acad Sci USA 110:10039–10044. doi:10.1073/pnas.1215283110. 23630264 PMC3683749

[4] Stettner AI, Segrè D. 2013. The cost of efficiency in energy metabolism. Proc Natl Acad Sci USA 110:9629–9630. doi:10.1073/pnas.1307485110. 23729810 PMC3683743

[5] Ameyama M, Shinagawa E, Matsushita K, Adachi O. 1981. D-Glucose dehydrogenase of *Gluconobacter suboxydans*: solubilization, purification and characterization. Agri Biol Chem 45:851–861.

[6] Lessie TG, Phibbs PV, Jr. 1984. Alternative pathways of carbohydrate utilization in pseudomonads. Annu Rev Microbiol 38:359–388. doi:10.1146/annurev.mi.38.100184.002043. 6388497

[7] Chavarría M, Nikel PI, Pérez-Pantoja D, de Lorenzo V. 2013. The Entner–Doudoroff pathway empowers *Pseudomonas putida* KT2440 with a high tolerance to oxidative stress. Environ Microbiol 15:1772–1785. doi:10.1111/1462-2920.12069. 23301697

[8] Olavarria K, Marone MP, da Costa Oliveira H, Roncallo JC, da Costa Vasconcelos FN, da Silva LF, Gomez JGC. 2015. Quantifying NAD(P)H production in the upper Entner–Doudoroff pathway from *Pseudomonas putida* KT2440. FEBS Open Bio 5:908–915. doi:10.1016/j.fob.2015.11.002. PMC4669411 26702395

[9] del Castillo T, Ramos JL, Rodríguez-Herva JJ, Fuhrer T, Sauer U, Duque E. 2007. Convergent peripheral pathways catalyze initial glucose catabolism in *Pseudomonas putida*: genomic and flux analysis. J Bacteriol 189:5142–5152. doi:10.1128/JB.00203-07. 17483213 PMC1951859

[10] Wilkes RA, Mendonca CM, Aristilde L. 2019. A cyclic metabolic network in *Pseudomonas protegens* Pf-5 prioritizes the Entner-Doudoroff pathway and exhibits substrate hierarchy during carbohydrate co-utilization. Appl Environ Microbiol 85:e02084-18. doi:10.1128/AEM.02084-18. 30366991 PMC6293094

[11] Eisenberg RC, Dobrogosz WJ. 1967. Gluconate metabolism in *Escherichia coli*. J Bacteriol 93:941–949. doi:10.1128/jb.93.3.941-949.1967. 5337840 PMC276539

[12] Olavarria K, De Ingeniis J, Zielinski D, Fuentealba M, Muñoz R, McCloskey D, Feist AM, Cabrera R. 2014. Metabolic impact of an NADH-producing glucose-6-phosphate dehydrogenase in *Escherichia coli*. Microbiology (Reading) 160:2780–2793. doi:10.1099/mic.0.082180-0. 25246670

[13] Zhao J, Baba T, Mori H, Shimizu K. 2004. Effect of *zwf* gene knockout on the metabolism of *Escherichia coli* grown on glucose or acetate. Metab Eng 6:164–174. doi:10.1016/j.ymben.2004.02.004. 15113569

[14] Volke DC, Olavarría K, Nikel PI. 2021. Cofactor specificity of glucose-6-phosphate dehydrogenase isozymes in *Pseudomonas putida* reveals a general principle underlying glycolytic strategies in bacteria. mSystems 6:e00014-21. doi:10.1128/mSystems.00014-21. 33727391 PMC8546961

[15] Lessmann D, Schimz KL, Kurz G. 1975. D-glucose-6-phosphate dehydrogenase (Entner-Doudoroff enzyme) from *Pseudomonas fluorescens*. Purification, properties and regulation. Eur J Biochem 59:545–559. doi:10.1111/j.1432-1033.1975.tb02481.x. 1257

[16] Anderson BM, Anderson CD. 1995. Purification and characterization of *Azotobacter vinelandii* glucose-6-phosphate dehydrogenase: dual coenzyme specificity. Arch Biochem Biophys 321:94–100. doi:10.1006/abbi.1995.1372. 7639541

[17] Fuhrer T, Fischer E, Sauer U. 2005. Experimental identification and quantification of glucose metabolism in seven bacterial species. J Bacteriol 187:1581–1590. doi:10.1128/JB.187.5.1581-1590.2005. 15716428 PMC1064017

[18] Fuentealba M, Muñoz R, Maturana P, Krapp A, Cabrera R. 2016. Determinants of cofactor specificity for the glucose-6-phosphate dehydrogenase from *Escherichia coli*: simulation, kinetics and evolutionary studies. PLoS One 11:e0152403. doi:10.1371/journal.pone.0152403. 27010804 PMC4807051

[19] Lessie TG, Wyk JCV. 1972. Multiple forms of *Pseudomonas multivorans* glucose-6-phosphate and 6-phosphogluconate dehydrogenases: differences in size, pyridine nucleotide specificity, and susceptibility to inhibition by adenosine 5'-triphosphate. J Bacteriol 110:1107–1117. doi:10.1128/jb.110.3.1107-1117.1972. 4402279 PMC247534

[20] Acero-Navarro KE, Jiménez-Ramírez M, Villalobos MA, Vargas-Martínez R, Perales-Vela HV, Velasco-García R. 2018. Cloning, overexpression, and purification of glucose-6-phosphate dehydrogenase of *Pseudomonas aeruginosa*. Protein Expr Purif 142:53–61. doi:10.1016/j.pep.2017.10.004. 28986240

[21] Basu A, Apte SK, Phale PS. 2006. Preferential utilization of aromatic compounds over glucose by *Pseudomonas putida* CSV86. Appl Environ Microbiol 72:2226–2230. doi:10.1128/AEM.72.3.2226-2230.2006. 16517677 PMC1393237

[22] Phale PS, Mohapatra B, Malhotra H, Shah BA. 2022. Eco-physiological portrait of a novel *Pseudomonas sp*. CSV86: an ideal host/candidate for metabolic engineering and bioremediation. Environ Microbiol 24:2797–2816. doi:10.1111/1462-2920.15694. 34347343

[23] Dhamale T, Saha B, Papade S, Singh S, Phale PS. 2022. A unique global metabolic trait of *Pseudomonas bharatica* CSV86^T^: metabolism of aromatics over simple carbon sources and co-metabolism with organic acids. Microbiology 168:e001206. doi:10.1099/mic.0.001206. 35925665

[24] Modak A. 2014. Studies on periplasmic GBP-dependent glucose ABC transporter from *Pseudomonas putida* CSV86. Indian Institute of Technology-Bombay, Mumbai, India.

[25] Choudhary A, Modak A, Apte SK, Phale PS. 2017. Transcriptional modulation of transport-and metabolism-associated gene clusters leading to utilization of benzoate in preference to glucose in *Pseudomonas putida* CSV86. Appl Environ Microbiol 83:e01280-17. doi:10.1128/AEM.01280-17. 28733285 PMC5601344

[26] Mohan K. 2018. Metabolic and molecular study of phenylpropanoid degradation and the carbon catabolite repression in *Pseudomonas putida* CSV86. Indian Institute of Technology-Bombay, Mumbai, India.

[27] Rowland P, Basak AK, Gover S, Levy HR, Adams MJ. 1994. The three–dimensional structure of glucose 6–phosphate dehydrogenase from *Leuconostoc mesenteroides* refined at 2.0Å resolution. Structure 2:1073–1087. doi:10.1016/S0969-2126(94)00110-3. 7881907

[28] Scopes R, Testolin V, Stoter A, Griffiths-Smith K, Algar E. 1985. Simultaneous purification and characterization of glucokinase, fructokinase and glucose-6-phosphate dehydrogenase from *Zymomonas mobilis*. Biochem J 228:627–634. doi:10.1042/bj2280627. 2992451 PMC1145031

[29] Hansen T, Schlichting B, Schönheit P. 2002. Glucose-6-phosphate dehydrogenase from the hyperthermophilic bacterium *Thermotoga maritima*: expression of the *g6pd* gene and characterization of an extremely thermophilic enzyme. FEMS Microbiol Lett 216:249–253. doi:10.1111/j.1574-6968.2002.tb11443.x. 12435510

[30] Cosgrove MS, Naylor C, Paludan S, Adams MJ, Levy HR. 1998. On the mechanism of the reaction catalyzed by glucose 6-phosphate dehydrogenase. Biochemistry 37:2759–2767. doi:10.1021/bi972069y. 9485426

[31] Cosgrove MS, Gover S, Naylor CE, Vandeputte-Rutten L, Adams MJ, Levy HR. 2000. An examination of the role of asp-177 in the His-Asp catalytic dyad of *Leuconostoc mesenteroides* glucose 6-phosphate dehydrogenase: X-ray structure and pH dependence of kinetic parameters of the D177N mutant enzyme. Biochemistry 39:15002–15011. doi:10.1021/bi0014608. 11106478

[32] Vought V, Ciccone T, Davino MH, Fairbairn L, Lin Y, Cosgrove MS, Adams MJ, Levy HR. 2000. Delineation of the roles of amino acids involved in the catalytic functions of *Leuconostoc mesenteroides* glucose 6-phosphate dehydrogenase. Biochemistry 39:15012–15021. doi:10.1021/bi0014610. 11106479

[33] Mercaldi GF, Dawson A, Hunter WN, Cordeiro AT. 2016. The structure of a *Trypanosoma cruzi* glucose-6-phosphate dehydrogenase reveals differences from the mammalian enzyme. FEBS Lett 590:2776–2786. doi:10.1002/1873-3468.12276. 27391210

[34] Petruschka L, Adolf K, Burchhardt G, Dernedde J, Jurgensen J, Herrmann H. 2002. Analysis of the *zwf*-*pgl*-*eda*-operon in *Pseudomonas putida* strains H and KT2440. FEMS Microbiol Lett 215:89–95. doi:10.1111/j.1574-6968.2002.tb11375.x. 12393206

[35] Maleki S, Hrudikova R, Zotchev SB, Ertesvag H. 2017. Identification of a new phosphatase enzyme potentially involved in the sugar phosphate stress response in *Pseudomonas fluorescens*. Appl Environ Microbiol 83:e02361-16. doi:10.1128/AEM.02361-16. 27836849 PMC5203634

[36] Daddaoua A, Krell T, Ramos JL. 2009. Regulation of glucose metabolism in *Pseudomonas*: the phosphorylative branch and Entner-Doudoroff enzymes are regulated by a repressor containing a sugar isomerase domain. J Biol Chem 284:21360–21368. doi:10.1074/jbc.M109.014555. 19506074 PMC2755860

[37] Leyn SA, Li X, Zheng Q, Novichkov PS, Reed S, Romine MF, Fredrickson JK, Yang C, Osterman AL, Rodionov DA. 2011. Control of proteobacterial central carbon metabolism by the HexR transcriptional regulator: a case study in *Shewanella oneidensis*. J Biol Chem 286:35782–35794. doi:10.1074/jbc.M111.267963. 21849503 PMC3195618

[38] Antunes A, Golfieri G, Ferlicca F, Giuliani MM, Scarlato V, Delany I. 2015. HexR controls glucose-responsive genes and central carbon metabolism in *Neisseria meningitidis*. J Bacteriol 198:644–654. doi:10.1128/JB.00659-15. 26644430 PMC4751820

[39] Lee WT, Levy HR. 1992. Lysine-21 of *Leuconostoc mesenteroides* glucose 6-phosphate dehydrogenase participates in substrate binding through charge–charge interaction. Protein Sci 1:329–334. doi:10.1002/pro.5560010304. 1304341 PMC2142207

[40] Opheim D, Bernlohr R. 1973. Purification and regulation of glucose-6-phosphate dehydrogenase from *Bacillus licheniformis*. J Bacteriol 116:1150–1159. doi:10.1128/jb.116.3.1150-1159.1973. 4148096 PMC246469

[41] Iyer RB, Wang J, Bachas LG. 2002. Cloning, expression, and characterization of the *gsd*A gene encoding thermophilic glucose-6-phosphate dehydrogenase from *Aquifex aeolicus*. Extremophiles 6:283–289. doi:10.1007/s00792-001-0255-2. 12215813

[42] Rauch B, Pahlke J, Schweiger P, Deppenmeier U. 2010. Characterization of enzymes involved in the central metabolism of *Gluconobacter oxydans*. Appl Microbiol Biotechnol 88:711–718. doi:10.1007/s00253-010-2779-9. 20676631

[43] Copley SD. 2020. Evolution of new enzymes by gene duplication and divergence. FEBS J 287:1262–1283. doi:10.1111/febs.15299. 32250558 PMC9306413

[44] Khanal A, Yu McLoughlin S, Kershner JP, Copley SD. 2015. Differential effects of a mutation on the normal and promiscuous activities of orthologs: implications for natural and directed evolution. Mol Biol Evol 32:100–108. doi:10.1093/molbev/msu271. 25246702 PMC4271523

[45] Geisbrecht BV, Gould SJ. 1999. The human PICD gene encodes a cytoplasmic and peroxisomal NADP^+^-dependent isocitrate dehydrogenase. J Biol Chem 274:30527–30533. doi:10.1074/jbc.274.43.30527. 10521434

[46] Jo SH, Son MK, Koh HJ, Lee SM, Song IH, Kim YO, Lee YS, Jeong KS, Kim WB, Park JW, Song BJ, Huh TL, Huhe TL. 2001. Control of mitochondrial redox balance and cellular defense against oxidative damage by mitochondrial NADP^+^-dependent isocitrate dehydrogenase. J Biol Chem 276:16168–16176. doi:10.1074/jbc.M010120200. 11278619

[47] Basu A, Dixit SS, Phale PS. 2003. Metabolism of benzyl alcohol *via* catechol *ortho*-pathway in methylnaphthalene-degrading *Pseudomonas putida* CSV86. Appl Microbiol Biotechnol 62:579–585. doi:10.1007/s00253-003-1305-8. 12687299

[48] Studier FW, Moffatt BA. 1986. Use of bacteriophage T7 RNA polymerase to direct selective high-level expression of cloned genes. J Mol Biol 189:113–130. doi:10.1016/0022-2836(86)90385-2. 3537305

[49] Mohapatra B, Nain S, Sharma R, Phale PS. 2022. Functional genome mining and taxono-genomics reveal eco-physiological traits and species distinctiveness of aromatic-degrading *Pseudomonas bharatica sp*. nov. Environ Microbiol Rep 14:464–474. doi:10.1111/1758-2229.13066. 35388632

[50] Kumar S, Stecher G, Tamura K. 2016. MEGA7: molecular evolutionary genetics analysis version 7.0 for bigger datasets. Mol Biol Evol 33:1870–1874. doi:10.1093/molbev/msw054. 27004904 PMC8210823

[51] Omotajo D, Tate T, Cho H, Choudhary M. 2015. Distribution and diversity of ribosome binding sites in prokaryotic genomes. BMC Genom 16:604. doi:10.1186/s12864-015-1808-6. PMC4535381 26268350

[52] Savli H, Karadenizli A, Kolayli F, Gundes S, Ozbek U, Vahaboglu H. 2003. Expression stability of six housekeeping genes: a proposal for resistance gene quantification studies of *Pseudomonas aeruginosa* by real-time quantitative RT-PCR. J Med Microbiol 52:403–408. doi:10.1099/jmm.0.05132-0. 12721316

[53] Pfaffl MW. 2001. A new mathematical model for relative quantification in real-time RT–PCR. Nucleic Acids Res 29:e45. doi:10.1093/nar/29.9.e45. 11328886 PMC55695

[54] Livak KJ, Schmittgen TD. 2001. Analysis of relative gene expression data using real-time quantitative PCR and the 2^−ΔΔCT^ methods. Methods 25:402–408. doi:10.1006/meth.2001.1262. 11846609

[55] Sambrook J, Russell DW. 2001. Directional cloning into plasmid vectors, p 84–87. *In* Molecular Cloning: A Laboratory Manual, 3rd ed, vol 1, Cold Spring Harbor Laboratory Press, New York.

[56] Bradford MM. 1976. A rapid and sensitive method for the quantitation of microgram quantities of protein utilizing the principle of protein-dye binding. Anal Biochem 72:248–254. doi:10.1006/abio.1976.9999. 942051

[57] Laemmli UK. 1970. Cleavage of structural proteins during the assembly of the head of bacteriophage T4. Nature 227:680–685. doi:10.1038/227680a0. 5432063

[58] Schägger H, von Jagow G. 1991. Blue native electrophoresis for isolation of membrane protein complexes in enzymatically active form. Anal Biochem 199:223–231. doi:10.1016/0003-2697(91)90094-a. 1812789

